# Improving information retrieval with multiple health terminologies in a quality-controlled gateway

**DOI:** 10.1186/2047-2501-1-8

**Published:** 2013-02-04

**Authors:** Lina F Soualmia, Saoussen Sakji, Catherine Letord, Laetitia Rollin, Philippe Massari, Stéfan J Darmoni

**Affiliations:** LITIS-TIBS EA 4108 & CISMeF Rouen University Hospital, Rouen, France

**Keywords:** Abstracting and indexing, Cataloguing, Information storage and retrieval, Internet, Terminology as topic, Vocabulary, Controlled

## Abstract

**Background:**

The Catalog and Index of French-language Health Internet resources (CISMeF) is a quality-controlled health gateway, primarily for Web resources in French (n=89,751). Recently, we achieved a major improvement in the structure of the catalogue by setting-up multiple terminologies, based on twelve health terminologies available in French, to overcome the potential weakness of the MeSH thesaurus, which is the main and pivotal terminology we use for indexing and retrieval since 1995. The main aim of this study was to estimate the added-value of exploiting several terminologies and their semantic relationships to improve Web resource indexing and retrieval in CISMeF, in order to provide additional health resources which meet the users’ expectations.

**Methods:**

Twelve terminologies were integrated into the CISMeF information system to set up multiple-terminologies indexing and retrieval. The same sets of thirty queries were run: (i) by exploiting the hierarchical structure of the MeSH, and (ii) by exploiting the additional twelve terminologies and their semantic links. The two search modes were evaluated and compared.

**Results:**

The overall coverage of the multiple-terminologies search mode was improved by comparison to the coverage of using the MeSH (16,283 *vs.* 14,159) (+15%). These additional findings were estimated at 56.6% relevant results, 24.7% intermediate results and 18.7% irrelevant.

**Conclusion:**

The multiple-terminologies approach improved information retrieval. These results suggest that integrating additional health terminologies was able to improve recall. Since performing the study, 21 other terminologies have been added which should enable us to make broader studies in multiple-terminologies information retrieval.

## Introduction

The Internet is fast becoming a recognized source of information in many fields, including health. In this context, several health gateways have been developed to support systematic resource discovery and help users find the health information they are looking for: quality-controlled subject portals were defined by Koch [1] as "Internet services which apply a comprehensive set of quality measures to support systematic resource discovery". These information seekers may be patients but also health professionals, such as physicians searching for clinical trials. Health gateways rely on thesauri and controlled vocabularies. Some of them are evaluated in [2]. Thesauri are a proven key technology for effective access to information since they provide a controlled vocabulary for indexing information. They therefore help to overcome some of the problems of free-text search by relating and grouping relevant terms in a specific domain.

In the framework of biomedical domain, several health portals could be rated as quality-controlled such as: Intute (UK), Health in site (AN) and CISMeF (Catalog and Index of French-language Health Internet resources) designed to catalogue and index the most important sources of institutional health information in French (n=89,751). Doc’CISMeF [3] is the search tool associated to CISMeF. It was designed to provide the most relevant resources not only for health professionals and medical students, but also for patients, their families, and the cyber-citizens. We defined Internet resources as Internet Web sites and Web documents obtained from these latter resources. Between 1995 and 2005, CISMeF used two standards to organize information: the MeSH (Medical Subject Headings) thesaurus [4] (used to index the scientific articles of the MEDLINE bibliographic database) and the Dublin Core meta-data set [5].

To evaluate the catalogue and to ensure its continuous relevance, several studies and improvements have been carried out, in order to provide users with the best information. Many tools have been developed: they exploit techniques such as natural language processing, statistics, lexical and background knowledge [6, 7], the structure of the MeSH thesaurus [8], but also simple spelling-correctors based on edit distances [9].

Faced with a growing amount of online resources to be indexed and included in the catalogue, the CISMeF team consistently evaluated manual and advanced automatic MeSH indexing techniques. As it is difficult for a single terminology to reflect the entire health domain in its different contexts, in 2008 the CISMeF team made possible the application of automated indexing using several health terminologies to "low priority resources". For that purpose, the F-MTI (French Multi-Terminological Indexer) tool was developed, and used to index health resources in CISMeF [10]. In addition to the MeSH thesaurus, four health terminologies were included: ICD-10 (International Classification of Diseases), SNOMED 3.5 (Systematized Nomenclature of Medicine), CCAM (the French equivalent of US CPT) and TUV (a French terminology for therapeutic and clinical indications for the use of drugs). In 2009, another study was performed [11] to evaluate the added value of multi-terminology indexing using the F-MTI in order to automatically index CISMeF resources. The study presented the efforts to assess the added value of integrating four new terminologies (Orphanet, ATC, drug names, MeSH supplementary concepts) into F-MTI’s knowledge sources and performing the automatic indexing on the online health resources’ titles and abstracts. The performance of F-MTI including five health terminologies on CISMeF manually-indexed resources with only the title was 25.9% precision and 13.5% recall, while the performance with nine terminologies was 27.9% precision (+2%) and 19.7% recall (+6.2%). The use of nine terminologies instead of five terminologies allowed the improvement of CISMeF web resources indexing.

After these first experiences on exploiting multiple terminologies for automatic indexing, we attempt to go further and evaluate information retrieval founded on multiple terminologies in terms of coverage and precision in the CISMeF catalog (*vs.* mono-terminology information retrieval). The use of multiple terminologies is recommended to increase the number of the lexical and graphical forms of a biomedical term recognized by a search engine. For this reason, CISMeF evolved recently from a mono-terminology approach using the MeSH main headings and subheadings to a multiple terminologies paradigm using, in addition to the MeSH thesaurus, vocabularies and classifications that deal with various aspects of health.

## Methods

### Multiple-terminologies version of the CISMeF information system

The need for the shift from a mono-terminological world (restricted to the MeSH thesaurus for indexing and retrieval) to a multiple-terminological universe (based on several heath terminologies) is felt more and more by the fact that each terminology not only has different objectives and context usage, but also attempts to overcome the potential imperfections of the MeSH thesaurus (for indexing and searching information). Indeed, according to the usage context, certain terminologies can be more suitable than others. For example, a pharmacist, probably, prefers to use the ATC (Anatomical Therapeutic Chemical) classification or a CAS code to have more specific information about drugs. In contrast, a medical student would use the MeSH thesaurus to obtain the expected bibliographical documents. Therefore, the multiple-terminologies version of the CISMeF information system was performed by integrating the main health terminologies available in French into a single structure. In addition to the MeSH thesaurus, many French (or their French translation) terminologies (n=12) have been added, namely SNOMED (Systematized Nomenclature of MEDicine) [12], ICF (International Classification of Functioning, the handicap and health) [13], ICD-10 (International Classification of the Diseases, version10) [14], CCAM (Common Classification of the Medical Procedures) [15], ICPC2 (International Classification of the Primary Care, second edition) [16], DRC (Consultation Results Dictionary) [17], ATC (Anatomical Therapeutic Chemical) classification [18], MedDRA (Medical Dictionary for Regulatory Activities) [19], MedlinePlus [20], WHO-ART (WHO Adverse Reactions Terminology) [21] and several French codes related to drugs. Most of these terminologies (n=9) are present in the UMLS [22] (Unified Medical Language System) Metathesaurus (for example MeSH, SNOMED, ICD-10) and some (n=3) are not (for example CCAM, DRC). In the health domain, the UMLS project is the research program launched by the US National Library of Medicine to establish knowledge sources in order to facilitate the development of systems which help health professionals to obtain biomedical information. The knowledge sources can be employed to establish interoperability between the heterogeneous information systems and to solve the problems of the integration of several terminologies due to their differences. The UMLS knowledge sources are the Metathesaurus, the Semantic Network and the Specialist Lexicon, a medical lexicon.

Our objective must take into account the availability of these medical terminologies, classifications, thesaurus and nomenclatures in French and of the existing mapping between them to insure their interoperability. To allow semantic expansion in information retrieval algorithm, several semantic harmonizations were carried out [23], such as:

(i) Conceptual mapping via the UMLS’s Metathesaurus. All the terminologies available in the UMLS Metathesaurus are mapped together thanks to the same concept identifier with the same CUI (Concept Unique Identifier) of concepts (an exact match);
(ii) Manual mapping between terminologies: e.g. MeSH-CCAM; MeSH-ATC;
(iii) Automatic mapping using Natural Language Processing (NLP) methods developed: e.g. Orphanet-MeSH.

### Multiple-terminologies model

In order to establish a generic and uniform model gathering all these terminologies in CISMeF information system, we had to take into account their original formats (SQL format, database, XML …etc.) to model, later on, each one by generating their RDF [24] (Resource Description Format) format in the purpose to have a homogeneous database. Thus, we joined together, in the same structure, terminologies, thesauri, nomenclatures and classifications, having particular specificities without loosing of any information. The model is described in [25] and it is centered on the "Descriptor" entity which includes all the terms which can describe the terminologies concepts. This class defines the common terminologies’ attributes. The specific attributes are represented by another entity which makes it possible to keep the entire information of each terminology. The definitions of the descriptors are multilingual and of different types. To allow enrichment of the user query and without being regarded as index terms, synonyms are represented in the model and added to the database. Each descriptor belongs to one of the integrated terminologies. These latter are also represented in the model. Hierarchical relations and intra-terminological non-hierarchical relations within the same terminology and inter-terminological relationships connecting terminologies between them, as it is performed with the semantic network and the meta-thesaurus of UMLS, are also represented in the model.

### Multiple-terminologies information retrieval algorithm

The information retrieval algorithm described in [26] was adapted to the multiple-terminologies universe in order to generalize the search process with all the new terminologies integrated in the CISMeF information system.

#### Query process

User query was segmented on words and insignificant terms (stop words such as *the*, *a, I*) were eliminated. Then, with the list of the most important words of the user query, the bag of words algorithm [26] was performed to recognize the best descriptors belonging to the different terminologies available in the CISMeF information system and then the next stage was to build the Boolean query to be performed on Doc'CISMeF. For example, after eliminating insignificant words (of, the) from the user query « *disease of the digestive system* », the bag of words obtained was {*disease; digestive; system*}. Then, the identification of the terminologies’ descriptors revealed « *Digestive System Diseases* » which is a MeSH descriptor, « *Digestive diseases* » which is a MedlinePlus descriptor, « *Diseases of the digestive system* » which is an ICD-10 descriptor and « *Disease of digestive system, nos* » which is a SNOMED descriptor. The resulting Boolean query was: (Digestive System Diseases.mr[MSH]) OR (Digestive diseases.mr[MED] and system.ti) or (Diseases of the digestive system.mr[ICD]) OR (Disease of digestive system, nos.mr[SNO]), with *mr:* the term represents a descriptor; *ti:* the term is present in the title; *ICD:* the term is included in the ICD-10; *MSH:* the term is a MeSH descriptor; *SNO:* the term is included in SNOMED-CT; *MED:* the term is included in MedlinePlus.

#### Information retrieval algorithm

The multiple-terminologies information retrieval algorithm is based on bag of words and has the same three steps of the mono-terminology information retrieval algorithm [26] which consists of the following steps:

(i) Searching at the level of the resources’ titles or in the resources indexing terms, (ii);
(ii) If (i) provides 0 results, searching on the resources metadata (e.g. author, date, editors, resources description …etc.);
(iii) If (ii) provides 0 results, searching in full text of the resources.

The result of the information retrieval was enriched with the resources indexed by the terms subsuming (directly or indirectly) (for example « *Digestive system fistula* », « *biliary tract diseases* », « *digestive system abnormalities* » …etc.) the identified terminologies descriptors. This option can be excluded when the user prefers a restricted result.

#### Sample test

Multiple-terminologies information retrieval was evaluated on a CISMeF corpus of 37,263 manually indexed web resources and 5,059 automatically indexed comprised of at least a title and a subtitle out of a total of 35,764 automatically indexed web resources. For each manual indexed resource in the corpus, the indexers selected the title, the subtitle and wrote a short abstract which summarizes the web resource meaning. They also described and indexed the resource by selecting a set of terms (descriptors) belonging to the medical terminologies available in the CISMeF information system.

For the automatically indexed resources, the process is done thanks to the bag of words algorithm [26]. It provides the different descriptors of the different terminologies, describing the resources’ content. For each resource, first the title/subtitle is broken into sentences. Then each sentence is normalized (accents are removed, all words are switched to lower case and stemmed etc.) and stop words are removed to form a bag of words of the most significant words. The “bag” thus obtained is matched against all the terminologies available in CISMeF information system. All terminologies’ terms containing at least one word of the sentence are retrieved. Longer matches are preferred to shorter ones. For example, indexing a web resource by “*Choroid neoplasms*” is considered more precise than “*Neoplasms*”, when the constituted bag of words contains, among others, “*choroid*, *neoplasm*, *cancer* …etc.”

### Evaluation

In order to evaluate the potential added value of the multiple-terminologies universe, a set of queries was defined, mainly based on the logs of the Doc’CISMeF search engine. This set of queries was (i) first launched (according to the algorithm described above) with only the MeSH thesaurus, (ii) then with all CISMeF terminologies and (iii) finally with all terminologies *except* the MeSH thesaurus *((ii)-(i))*. The evaluation was performed on the disparate resources, restituted by the third step (iii). For the purpose of the study, ten queries with one term, eleven queries with two terms and eleven queries with three terms were chosen which potentially produces different results between the mono-terminological search (using only MeSH) and multiple-terminologies search. These queries are listed in Table 1. To measure the potential added value of using several terminologies, CISMeF information retrieval algorithm was tested to evaluate the mapping between the resources multiple-terminologies indexing terms and the user queries. For evaluation, human experts were chosen as the gold standard. Three different experts performed the evaluation: (a) one CISMeF indexer, who is a pharmacist and librarian (CL), (b) one senior physician in intensive care (PM) and (c) one junior physician in occupational medicine (LR). The role of these domain experts is to judge the relevance of the disparate resources between the multiple-terminologies search mode, using all the medical terminologies, and the mono-terminology search mode using only the MeSH thesaurus. The results were rated by the three experts as (i) *good* if the resource was in perfect concordance with the required topic, (ii) *bad* if it generated more noise than precision or otherwise (iii) *intermediate*. For each type of query (one-word query, two-words query and three-words query), each expert evaluated the relevance of an identical set of additional web resources retrieved by the multiple-terminologies approach (according to each user query, the number of the evaluated web resources was ranged between 1 and 268).

**Table 1:** **Sets of 1-word, 2-words and 3-words queries**

**1-word queries**	Otitis
	Asthma
	Embolism
	Hypertension
	Rheumatism
	Spine
	anti-hypertensors
	pain
	ulcer
	endoscopy
**2-words queries**	pathological anatomy
	locomotor apparatus
	malformatives uropathies
	nutritional evaluation
	breathing apparatus
	physiology blood
	child development
	administration pharmacy
	parasitic diseases
	orthopedic surgery
	pancreatic hormones
**3-words queries**	urinary fecal incontinence
	gynecological surgical operation
	digestive system disease
	child psychomotor development
	breast cancer treatment
	pulmonary surgical operation
	vascular surgical operation
	neurological diseases
	file care male nurses
	nasal fossae anatomy

Another experimentation was performed on 20 "general" terms which correspond to medical specialties. Those terms are positioned on Top hierarchies such as "*Cardiology*", "*Surgery*", and “*Oncology*" …etc. For this second test, a physician has evaluated the relevance of the first 20 returned resources, because it is established that 95% of the end-users do not go beyond when using a general or a specialized search tool.

## Results

The first column of the Table 2 highlights the number of resources by mono-terminology search mode for each type of query. The second column enumerates the number of resources by multiple-terminologies search mode. The third column summarizes the difference as percentage between the two search modes. The highest percentage is detected for two-words queries (44.88%). Overall, the added-value of multiple-terminologies information retrieval in terms of the coverage was (+15%).

**Table 2:** **Number of obtained resources according to search modes and types of queries**

Query type	Single-terminology search	Multiple-terminologies search	Δ
1-word queries	2,942	3,432	+16.65%
2-words queries	3,353	4,858	+44.88%
3-words queries	7,864	7,993	+01.64%
Total	14,159	16,283	+15%

Table 3 shows the evaluation of the three raters, which was defined as the gold standard. Their evaluation focused on resources which were retrieved using the multiple-terminologies algorithm and not found by the mono-terminology approach. The values represent the percentages of the resources which were judged by the three human raters as good, intermediate or bad result, accounting for each type of query (one-word, two-words or three-words queries) and each evaluator. For one-word queries, the overall relevant result was rated at 68.2%, whereas the intermediate result was 10.4% and the irrelevant result was 21.4%. For two-words queries, the global result was slightly different insofar as the best percentage was always judged as good (57.8%) but the percentage which followed was concerned with the intermediate result (31.5%) and finally, the irrelevant result with 10.7%. For three-words queries, the relevant result was rated at 43.7%, the intermediate result at 32.4% and the irrelevant result at 23.9%. Overall, the average of results according to the three types of queries are displayed in Table 4: the first expert rated good results in 53.8% of the cases, the second expert in 68.3% of the cases and the third expert in 47.7% of the cases. There is a statistical difference between the result’s relevance judged by the three raters for each kind of queries (Chi 2 test, p<0.0001) and for the aggregated results (Chi 2 test, p<0.0001).

**Table 3:** **Evaluation results of information results with multiple-terminologies mode for each set of queries**

Query type	1-word queries			2-words queries			3-words queries		
**Evaluation**	**G (%)**	**I (%)**	**B (%)**	**G (%)**	**I (%)**	**B (%)**	**G (%)**	**I (%)**	**B (%)**
Expert 1	73.0	05.3	21.7	47.2	33.2	19.6	41.1	21.4	37.5
Expert 2	75.0	04.8	20.2	76.0	18.6	5.4	53.9	41.0	05.1
Expert 3	56.7	20.9	22.4	50.3	42.7	07.0	36.0	34.9	29.1
Average	**68.2**	10.4	21.4	**57.8**	31.5	10.7	**43.7**	32.4	23.9

G: good, I: intermediate and B: bad.

**Table 4:** **Evaluation results of information retrieval with multiple-terminologies mode for the whole set of queries**

Evaluation	Good (%)	Intermediate (%)	Bad (%)
Expert 1	53.3	20.0	26.3
Expert 2	68.3	21.5	10.2
Expert 3	47.7	32.8	19.5
Average	**56.6**	54.7	18.7

For the second experimentation with 20 general queries, Table 5 shows the number of retrieved resources with a single vs. multiple terminologies. An average of 17% supplementary resources are retrieved using multiple terminologies and confirms the first results of the Table 2. For each general query, the relevance of the top 20 returned resources was evaluated (using good, intermediate and bad rates). The results are displayed in the Table 6. Due to some broken links 11 resources among the 400 returned were not evaluated. However, 70.44% were rated as Good by a physician, 21.07% as intermediate and only 8.48% as bad.

**Table 5:** **Number of resources retrieved by 20 "general" queries with a single terminology vs. multiple terminologies**

General query	Single terminology	Multiple terminologies	Δ
Diagnosis	13,132	13,482	+02.66%
Toxicology	11,980	12,462	+04.02%
Neurology	9,325	11,493	+23.24%
Infectious Diseases	6,557	9,130	+39.24%
Pediatrics	7,560	251	+03.32%
Cardiology	5,288	2,388	+45.15%
Oncology	5,626	1,063	+18.89%
Surgery	5,504	320	+05.81%
Rheumatology	4,408	856	+19.42%
Gastroenterology	4,069	1,106	+27.18%
Allergies and Immunology	4,598	573	+12.46%
Metabolism	3,797	849	+22.36%
Dermatology	3,196	1,427	+44.64%
Nutrition	3,455	1,027	+29.72%
Pneumology	3,466	584	+16.84%
Gynecology	3,186	850	+26.68%
Hematology	2,906	1,075	+36.99%
Endocrinology	3,168	666	+21.02%
Obstetrics	3,063	316	+10.31%
Virology	3,122	257	+08.23%
Total	107,406	19,181	+17.86%

**Table 6:** **Relevance of the 20 top resources retrieved for 20 general queries using multiple terminologies mode (G: Good, I: Intermediate, B: Bad)**

General query	Relevance		
	G	I	B
Diagnosis	15	2	0
Toxicology	20	0	0
Neurology	8	4	8
Infectious Diseases	20	0	0
Pediatrics	13	4	2
Cardiology	18	0	1
Oncology	18	1	0
Surgery	15	0	5
Rheumatology	9	8	3
Gastroenterology	20	0	0
Allergies and Immunology	2	17	1
Metabolism	18	2	0
Dermatology	16	4	0
Nutrition	19	1	0
Pneumology	12	7	0
Gynecology	19	1	0
Hematology	3	10	7
Endocrinology	11	9	0
Obstetrics	12	1	5
Virology	6	11	1
Total*	274 70.44%	82 21.07%	33 08.48%

*due to some broken links several (11 among 400) resources were not evaluated.

## Discussion

The results of this study indicated that the multiple-terminologies mode retrieved resources that were not retrieved by mono-terminology mode. In fact, the added-value of the multiple-terminologies information retrieval in terms of the coverage was +15% for the first run of the method (16,283 resources provided by multiple-terminologies search mode vs. 14,159 by the mono-terminology search mode) and +17% for the second run of the methods on general queries. This can improve health information retrieval in CISMeF or any portal such as PubMed and, in general, in any catalogue or portal based on multiple-terminologies such as National Guideline Clearinghouse (NGC, URL: http://www.guideline.gov/) which, recently, has also shifted to a multiple-terminologies approach (URL: http://www.guideline.gov/content.aspx?id=15096&search=pain).

Therefore, after this evaluation, the results were considered by the CISMeF team to be sufficient to implement multiple-terminologies information retrieval algorithm in the Doc’CISMeF search engine (as an optional choice). For example, for the query “*spine*” the mono-terminology information retrieval algorithm provided 213 resources on April 2012 (URL: http://doccismef.chu-rouen.fr/servlets/Simple?Mot=rachis&aff=4&tri=20&datt=1&cis=cis&msh=msh&pha=pha&debut=0) and multiple-terminologies information retrieval algorithm provided 238 resources (URL: http://doccismef.chu-rouen.fr/servlets/Simple?Mot=rachis&aff=4&tri=20&datt=1&atc=atc&cca=cca&cif=cif&cim=cim&cip=cip&cis=cis&cla=cla&drc=drc&fma=fma&lpp=lpp&mdr=mdr&med=med&msh=msh&ncc=ncc&orp=orp&pha=pha&sno=sno&uni=uni&vcm=vcm&art=art&wps=wps&toutes=toutes&debut=0). The results show that in spite of discrepancies between the experts’ ratings, the global result is quite interesting, as good results for the three experts were respectively 53.8%, 68.3% and 47.7% (see Table 4). In general, the average of the results is classified as follows: good results (56.6%) are in the top, followed by the intermediate results (24.7%) and lastly the bad ones (18.7%). The difference between the resources of the mono-terminology search and the multiple-terminologies search is less significant for three-words queries due to the difficulty of finding a correlation between user query and the multiple-terminologies indexing terms. For example, it is more difficult to have a good mapping between the user query “*treatment of the breast cancer*” and the resource index because there is no descriptor belonging to any terminology of CISMeF information system which expresses this query. For the second run of the method, 70.44% of the 20 top returned resources were rated as having a good relevance.

In contrast, to highlight the add-value of our approach, let us consider the user query “*mrkh*” which provides a better result with the multiple-terminologies information retrieval algorithm in comparison to the mono-terminology information retrieval due to the fact that the term *"mrkh"* does not belong to the MeSH thesaurus. Indeed, we created a CISMeF synonym *"mrkh"* for the MedDRA term “*Mayer-rokitansky-kuster-hauser syndrome*”, and then we linked the two terms in order to have semantic interoperability between health terminologies. Therefore, using both terms was more efficient for information retrieval process.

The limitation of the study was the number of the evaluated queries. Thus, the established study constitutes a proof of the concept of the proposed model and its implementation. The integration of new medical terminologies in CISMeF (for example the Foundational Model of Anatomy or the Human Phenotype Ontology) and the improvement of resource indexing (manual and automatic) would permit a broader study and certainly obtain more meaningful results.

In addition, considering the limited knowledge of the indexers concerning the new terminologies integrated in CISMeF, the rate of manual indexing by multiple terminologies was still rather low compared with that performed by only the MeSH thesaurus. Nonetheless, 5,164 manually indexed resources out of 37,263 (13.8%) are already being indexed with at least one terminology besides the MeSH (ATC (n=4616), CCAM (n=326) and SNOMED (n=39) etc.), mainly with the ATC for the creation of the PSIP Drug Information Portal [27].

To the best of our knowledge, this study was the first which evaluated multiple-terminologies information retrieval in any health site. This multiple-terminologies information retrieval approach could be applied to any Web portal currently using the MeSH and in particular to MEDLINE/PubMed as newly included citations are now automatically indexed with MetaMap [28], which provides multiple-terminologies indexing.

In conclusion, the strategic decision of the CISMeF team has made possible the evolution from a mono-terminological world to a multiple-terminological universe through the integration of the main health terminologies available in French in the CISMeF information system. The contribution of this new universe is to overcome the relative weakness of the MeSH thesaurus and to improve health information retrieval.

## Abbreviations

CISMeF: Catalog and Index of French-language Health Internet resources
MeSH: Medical Subject Headings
MEDLINE: MEDical Literature Analysis and Retrieval System on LINE
F-MTI: French Multi-Terminological Indexer
ICD-10: International Classification of Diseases
SNOMED: Systematized Nomenclature of Medicine
CCAM: Common Classification of the Medical Procedures
ATC: Anatomical Therapeutic Chemical
CAS: Chemical Abstract Service
MedDRA: Medical Dictionary for Regulatory Activities
WHO-ART: WHO Adverse Reactions Terminology
UMLS: Unified Medical Language System.
